# Elevated Glucose and Insulin Levels Decrease DHA Transfer across Human Trophoblasts via SIRT1-Dependent Mechanism

**DOI:** 10.3390/nu12051271

**Published:** 2020-04-30

**Authors:** Jay S. Mishra, Hanjie Zhao, Sari Hattis, Sathish Kumar

**Affiliations:** 1 Department of Comparative Biosciences, School of Veterinary Medicine, University of Wisconsin, Madison, WI 53706, USA; jay.mishra@wisc.edu (J.S.M.); hzhao274@wisc.edu (H.Z.); hattis@wisc.edu (S.H.); 2 Department of Obstetrics and Gynecology, School of Medicine and Public Health, University of Wisconsin, Madison, WI 53792, USA

**Keywords:** gestational diabetes, DHA, fatty acid transport, lipid metabolism, CD36, FABP3, FABP4, SIRT1, trophoblast

## Abstract

Gestational diabetes mellitus (GDM) results in reduced docosahexaenoic acid (DHA) transfer to the fetus, likely due to placental dysfunction. Sirtuin-1 (SIRT1) is a nutrient sensor and regulator of lipid metabolism. This study investigated whether the high glucose and insulin condition of GDM regulates DHA transfer and expression of fatty acid transporters and if this effect is related to SIRT1 expression and function. Syncytialized primary human trophoblasts were treated with and without glucose (25 mmol/L) and insulin (10^−7^ mol/L) for 72 h to mimic the insulin-resistance conditions of GDM pregnancies. In control conditions, DHA transfer across trophoblasts increased in a time- and dose-dependent manner. Exposure to GDM conditions significantly decreased DHA transfer, but increased triglyceride accumulation and fatty acid transporter expression (CD36, FABP3, and FABP4). GDM conditions significantly suppressed SIRT1 mRNA and protein expression. The SIRT1 inhibitor decreased DHA transfer across control trophoblasts, and recombinant SIRT1 and SIRT1 activators restored the decreased DHA transport induced by GDM conditions. The results demonstrate a novel role of SIRT1 in the regulation of DHA transfer across trophoblasts. The suppressed SIRT1 expression and the resultant decrease in placental DHA transfer caused by high glucose and insulin levels suggest new insights of molecular mechanisms linking GDM to fetal DHA deficiency.

## 1. Introduction

Gestational diabetes mellitus (GDM) is a common metabolic disorder affecting 5–20% of all pregnancies, depending on the diagnosis method and ethnicity [1]. GDM is characterized by glucose intolerance diagnosed for the first time during pregnancy caused due to reduced responsiveness to insulin [2]. This insulin resistance (IR) induces alterations in lipid metabolism leading to dyslipidemia in GDM women [3]. GDM increases perinatal morbidity and increases the risk of developing type 2 diabetes mellitus later in life [4,5,6]. Moreover, children born to mothers with poor gestational glucose control suffer neurobehavioral and cognitive dysfunction compared to children born to non-diabetic women [7,8].

Docosahexaenoic acid (DHA, 22:6 n − 3) is a long-chain polyunsaturated fatty acid that is essential for neurogenesis and brain development during the early stages of fetal life [9,10]. The neurodevelopmental dysfunctions observed in children of mothers with GDM is suggested to be as a result of lower DHA transfer from the mother to the fetus [11,12]. Maternal DHA is the main source of DHA for the fetus, as the fetus and placenta have little or no ability to synthesize DHA [13]. The placenta can preferentially uptake DHA from the maternal circulation and transfer it to the fetus, and this is shown by higher DHA levels in cord blood than maternal circulation [14].

Seven out of nine case-controlled studies show that cord blood levels of DHA are lower in GDM pregnancies than in non-diabetic pregnancies [15,16,17,18,19,20,21,22,23]. Specifically, maternal DHA levels are found to be 11–44% higher in GDM, while cord blood levels are lower compared to non-diabetic pregnancies [24]. Supplementation of DHA in GDM women increased DHA in maternal plasma but failed to increase DHA in cord blood [25]. Collectively, these observations indicate reduced transplacental transport of DHA to the fetus in GDM. The exact mechanism for the reduced placental transport of DHA in GDM pregnancies is not clear. Specifically, how IR affects DHA transport across syncytiotrophoblasts, the primary barrier between maternal and fetal circulations, is not well understood. The transport of fatty acid through the syncytiotrophoblasts is regulated by many proteins, such as fatty acid transport proteins (FATP)-1-6, cluster of differentiation 36 (CD-36), and fatty acid-binding proteins (FABP) -1, 3, 4, 5, 7 [26]. In the GDM placenta, the expression of FATP-1 and FATP-4 were found to be decreased, while the expression of CD36 and FATP6 was increased compared to nondiabetic pregnancies [27].

Sirtuin1 (SIRT1) is a ubiquitously-expressed nutrient sensor, which closely correlates with lipid metabolism and metabolic fluxes [28,29]. The expression of SIRT1 is found to be downregulated in peripheral blood mononuclear cells of individuals with impaired glucose tolerance [30,31]. In vitro cell culture studies and animal experiments demonstrate that SIRT1 deficiency in hepatocytes induces lipid deposition and fatty liver disease [32,33]. Conversely, SIRT1 activation/overexpression protected against lipid accumulation and steatosis [34]. This suggests that the expression and function of SIRT1 directly relates to lipid metabolism. SIRT1 is highly expressed in trophoblasts [35], and Sirt1-null embryos are consistently growth-restricted [36,37,38]; however, its regulatory role in placental nutrient transport is yet to be studied. In this study, we investigated SIRT1’s role in placental nutrient transport, with a focus on transcellular DHA transport in trophoblasts from normal and insulin-resistant GDM conditions. We hypothesized that the high glucose and insulin condition of GDM regulates DHA transfer and expression of fatty acid transporters and that this effect is related to SIRT1 expression and function. This study, using primary human trophoblasts for the first time, shows that the GDM conditions induce the accumulation of triglycerides and increase the expression of fatty acid transporters. Yet, the DHA transfer across the trophoblasts was significantly reduced. The suppressed SIRT1 expression caused by GDM conditions plays a role in contributing to the decreased DHA transfer, as SIRT1 activation restored the decreased DHA transfer caused by GDM conditions. These findings provide new insights of molecular mechanisms linking GDM to fetal DHA deficiency.

## 2. Materials and Methods

### 2.1. Primary Human Trophoblast Culture

Isolated primary human trophoblast cells were purchased from ScienCell (Carlsbad, CA, USA). The cells were cultured in proprietary trophoblast medium with fetal bovine serum, growth supplements, and antibiotics (penicillin/streptomycin) (ScienCell) in a humidified incubator under a 5% CO_2_ atmosphere at 37 °C. Trophoblasts were seeded at a density of 0.5 × 10^6^ cells/cm^2^ and 0.1 × 10^6^ cells/cm^2^ in 6-well plates or 12-well Transwell plates coated with human placental collagen (0.01% *w*/*v*). The medium was replenished every day for seven days to allow syncytialization of cells, which was confirmed by hPL and hCG mRNA expression and secretion of hCG, as described previously [39,40]. The insulin-resistant GDM trophoblast cell model was established by treating day four culture with 25-mmol/L glucose and 10^−7^-mol/L insulin for 72 h, as described previously [41]. Control cells were cultured with physiological glucose concentrations (5 mmol/L) and no insulin. The development of IR was evaluated by examining the mRNA expression of IR-related genes (IRS-1, IGF-1/2 and leptin). Changes in DHA transport across trophoblasts, triglyceride content, and fatty acid transporter mRNA and protein expression were measured and compared between cells cultured under standard (control) and high glucose and insulin conditions (referred as GDM conditions). 

### 2.2. Transport of DHA across Trophoblasts

The transcellular DHA transport was assessed using ^14^C-DHA (Moravek Inc., Brea, CA, USA) in trophoblasts grown on Transwell inserts. ^14^C-DHA was added to the apical compartment (“maternal side”), and their transfer across the trophoblast monolayer to the basal chamber (“fetal side”) was measured by collecting medium samples from the basal chamber at specific time points. The samples were mixed with 5 mL of the scintillation cocktail (PerkinElmer, Waltham, MA, USA), and radioactivity was measured using a Tri-carb 2100TR Liquid Scintillation Counter (PerkinElmer, Waltham, MA, USA). Trans-epithelial electrical resistance values and barrier integrity of trophoblast layers were monitored before and after the experiment using a Millicell ERS-2 Volt-Ohm Meter (Millipore, Bedford, MA, USA) [40]. The permeability properties across the trophoblast monolayer were evaluated by measuring the passage of the fluorescence dye Lucifer yellow (Sigma, Saint Louis, MO, USA) [40]. In some experiments, the cells were treated with SIRT1 inhibitor (EX527, 1 µmol/L), recombinant SIRT1 (500 ng/L), or SIRT1 activator (SIRT1729, 1 µmol/L) for 24 h, and then the ^14^C-DHA transfer assay was done. 

### 2.3. Quantification of Triglycerides

Intracellular triglyceride levels were quantified using a colorimetric assay that is based on enzymatic hydrolysis of the triglycerides to produce glycerol and free fatty acids, following manufacturer’s instructions (Cayman Chemical, Ann Arbor, MI, USA). The triglyceride content was normalized to the protein content that was measured using the BCA protein assay kit (Pierce; Thermo Scientific, Waltham, MA, USA). 

### 2.4. Quantitative Real-Time PCR (qRT-PCR) 

Total RNA was extracted as per the manufacturer’s instructions (RNeasy mini kit, Qiagen, Valencia, CA, USA). RNA integrity and quantity were determined using a DS-11 spectrophotometer (DeNovix, Wilmington, DE, USA). Total RNA (1 µg) was reverse transcribed using an iScript cDNA synthesis kit (Bio-Rad, Hercules, CA, USA). After dilution, cDNA corresponding to 100 ng of RNA was amplified by qRT-PCR using a CFX96 real-time thermal cycler (Bio-Rad) with Lightcycler Fast-Start DNA Sybr Green 1 master mix (Roche, Branford, CT, USA). Primers were designed to analyze genes related to IR: (IRS-1, IGF-1/2 and leptin); fatty acid transporters: FAT/CD36, FATP1, FATP2, FATP4, FATP6, FABP3, and FABP4; and SIRT1. Primer sequences are shown in Table 1. The 2^−ΔΔCT^ method was used for calculation, and the results were expressed as fold change of the gene of interest in treated versus control samples. All reactions were performed in duplicate, and GAPDH was used as an internal control.

### 2.5. Western Blotting

The cells were washed, scraped, harvested by microcentrifugation, and resuspended in an ice-cold RIPA buffer (Cell Signaling Technology, Danvers, MA, USA) containing a protease inhibitor tablet and phosphatase inhibitor cocktail-2 and -3 (Sigma). Cell lysates were centrifuged (14,000× *g* for 10 min at 4 °C), and the BCA assay kit (Pierce) was used to quantify protein concentration. The supernatant was resuspended in NuPAGE lithium dodecyl sulfate sample buffer and reducing agent (Invitrogen; Thermo Scientific, Waltham, MA, USA). Proteins (30 μg) were mixed with NuPAGE lithium dodecyl sulfate sample buffer and reducing agent (Invitrogen; Thermo Scientific) and resolved on 4–12% gradient NuPAGE Bis-Tris gels (Invitrogen) at 100 V for 2–3 h at room temperature alongside negative control and Precision Plus Standard (Kaleidoscope; Bio-Rad). After separation on the gel, proteins were transferred onto Immobilon-P membranes (Millipore, Billerica, MA, USA) at 100 V for 2 h. The membrane was blocked with 5% (wt/vol) nonfat dried milk for 1 h at room temperature. Blots were incubated overnight at 4 °C with respective primary antibodies against CD36, FABP3, FABP4, SIRT1 and β-actin (Cell Signaling Technologies or Sigma), and then for one hour with secondary antibody (anti-rabbit conjugated with horseradish peroxidase) prior to development with the Pierce ECL detection kits (Thermo Scientific). The densitometric analysis was done using Image J software. The results were expressed as ratios of the intensity of a specific band to that of β-actin. 

### 2.6. Data Analysis

GraphPad Prism was used for Statistical analyses (San Diego, CA, USA). Data are expressed as the mean ± SD of at least three independent experiments. The two groups were compared using unpaired Student *t*-tests. Multiple group comparisons were performed using ANOVA, followed by Newman–Keuls tests. Repeated measures of ANOVA were done for experiments performed over time. Statistically significant differences were reported when *p* < 0.05.

## 3. Results

### 3.1. Effect of GDM Conditions on the Expression of IR-Related Genes in Trophoblasts

**Figure 1:**
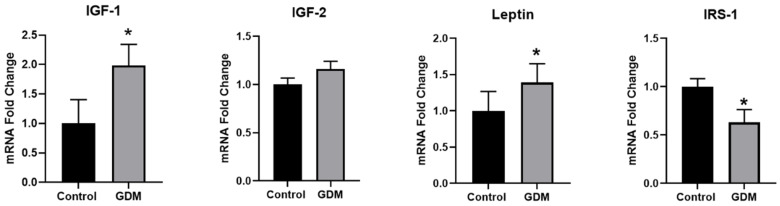
Changes in mRNA levels of genes related to insulin signaling in trophoblasts exposed to control and GDM conditions. Primary human trophoblasts were cultured in standard conditions or medium containing 25-mmol/L glucose and 10^−7^-mol/L insulin for 72 h, and real-time PCR was used to assess gene expression. Values are expressed as mean ± SD of three independent experiments. * *p* < 0.05 vs. control.

Consistent with previous studies [41], incubation of trophoblasts with glucose (25 mmol/L) and insulin (10^−7^ mol/L) caused IR, as evidenced by significant alterations (*p* < 0.05) in the expression of IR-related genes. Exposure to GDM conditions inhibited insulin signaling, as evidenced by the downregulation of IRS-1 (37% decrease) and upregulation of IGF-1 (2-fold increase) compared with the controls (Figure 1). The placenta is an important source of leptin during pregnancy. The results indicated that leptin expression was significantly increased in the IR trophoblasts (1.4-fold of the control), which could explain the high serum leptin level in GDM patients (Figure 1).

### 3.2. Effect of GDM Conditions on DHA Transport across Trophoblasts 

**Figure 2:**
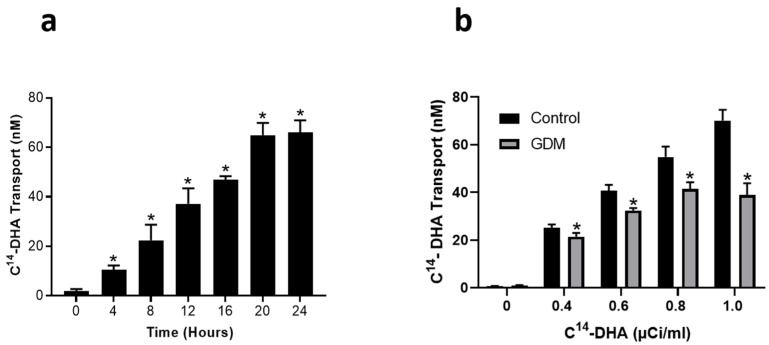
Changes in DHA transfer through the trophoblasts exposed to control and GDM conditions. Primary human trophoblasts were cultured in Transwell inserts in standard conditions or with medium containing 25-mmol/L glucose and 10^−7^-mol/L insulin for 72 h. Transfer of ^14^C-DHA from the apical to the basal chamber was measured using liquid scintillation counting. (**a**) Time-dependent increase in the transfer of ^14^C-DHA (1 μCi/mL) to the basal chamber. * *p* < 0.05 vs. zero time point. (**b**) Dose-dependent transfer of ^14^C-DHA to the basal chamber after 24 h. * *p* < 0.05 vs. respective time point in control. Values are expressed as mean ± SD of three independent experiments.

We next evaluated the rate of transport of DHA across the trophoblasts exposed to control and GDM conditions. In controls, ^14^C-DHA (1 µCi/mL) was found to transfer across the trophoblast monolayer in a time-dependent manner with a maximal effect observed at 24 h (*p* < 0.05) (Figure 2a). Furthermore, the ^14^C-DHA transfer rates across the trophoblasts increased with increasing concentrations of ^14^C-DHA added to the apical side (*p* < 0.05) (Figure 2b). In contrast, ^14^C-DHA transport rates across the trophoblasts were significantly reduced (*p* < 0.05) in the trophoblasts exposed to GDM conditions compared with trophoblasts maintained at physiological conditions (Figure 2b). GDM conditions decreased DHA transfer across trophoblasts by 15%, 23%, 28%, and 41% when 0.4, 0.6, 0.8 and 1.0 µCi ^14^C-DHA/mL were added to the apical side (Figure 2b).

### 3.3. Intracellular Triglyceride Levels

**Figure 3:**
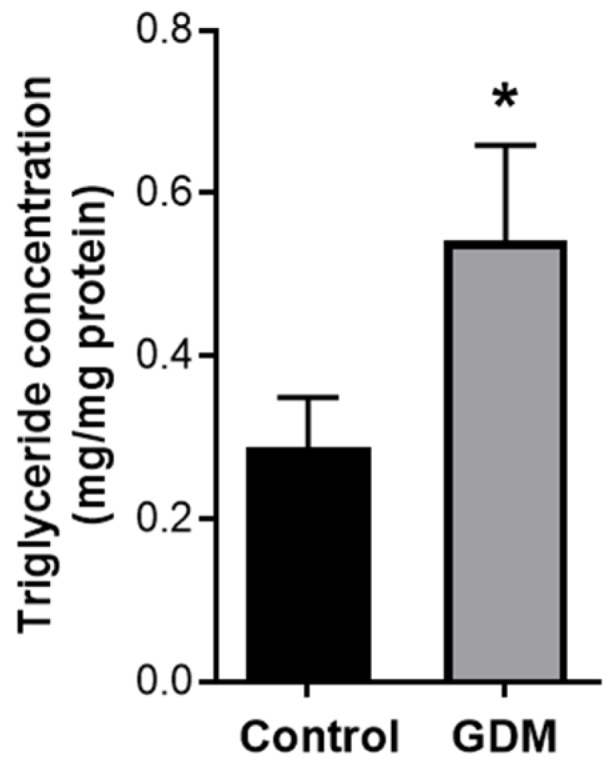
Changes in triglyceride content in the trophoblasts exposed to control and GDM conditions. Primary human trophoblasts were cultured in standard conditions or with medium containing 25-mmol/L glucose and 10^−7^-mol/L insulin for 72 h. The intracellular triglyceride level in the cells was quantified with a colorimetric assay. Values are expressed as means ± SD of three independent experiments. * *p* < 0.05 vs. control.

We then sought to confirm whether the lipid metabolism is altered in trophoblasts exposed to GDM conditions by assessing the effect on triglyceride content in trophoblasts. The data presented in Figure 3 shows that GDM conditions promote triglyceride accumulation in trophoblasts; levels were 2-fold greater (*p* < 0.05) in trophoblasts exposed to GDM conditions versus trophoblasts maintained at physiological conditions.

### 3.4. Expression of Fatty Acid Transport Genes in Trophoblasts

**Figure 4:**
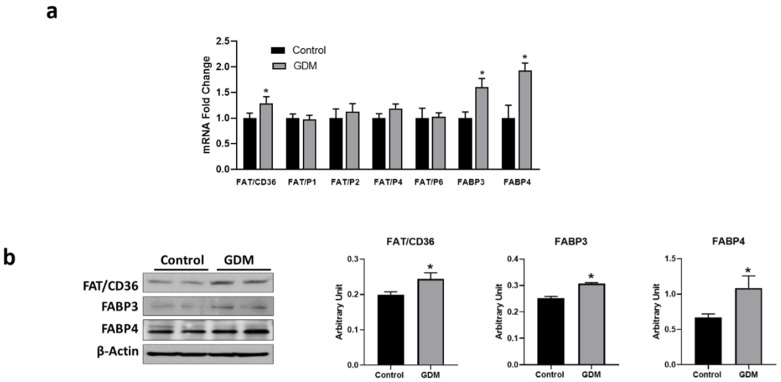
Changes in mRNA and protein levels of genes related to fatty acid transport in the trophoblasts exposed to control and GDM conditions. Primary human trophoblasts were cultured in standard conditions or with medium containing 25-mmol/L glucose and 10^−7^-mol/L insulin for 72 h. (**a**) Real-time PCR was used to assess mRNA levels, and quantitation of mRNA expression was normalized relative to GAPDH. (**b**) Western blotting was used for protein quantification. Representative blots for proteins are shown at the lefr; blot densities obtained from densitometric scanning of proteins normalized to β-actin are shown at the right. Values are expressed as mean ± SD of three independent experiments. * *p* < 0.05 vs. control.

Next, we investigated whether the GDM conditions-mediated decrease in DHA transfer and increase in intracellular triglyceride levels in trophoblasts was a consequence of an altered uptake or transport of fatty acids. To determine the effect of GDM conditions on fatty acid uptake and transport in trophoblasts, we compared mRNA expression of the key genes involved in these processes. The results showed that mRNA levels of genes for fatty acid uptake and transport, such as CD36, FABP3, and FABP4, were significantly increased in trophoblasts from GDM conditions (1.3-, 1.6-, and 1.9-fold, respectively) compared with controls (*p* < 0.05) (Figure 4a). Consistent with elevated mRNA levels, immunoblotting also revealed a significant increase in CD36 (1.2-fold), FABP3 (1.2-fold), and FABP4 (1.6-fold) protein in trophoblasts from GDM conditions compared with controls (*p* < 0.05) (Figure 4b). Protein expression of FABP3 and FABP4 in trophoblasts from GDM conditions were negatively correlated with DHA transport.

### 3.5. Effect of GDM Conditions on SIRT1 Expression

**Figure 5:**
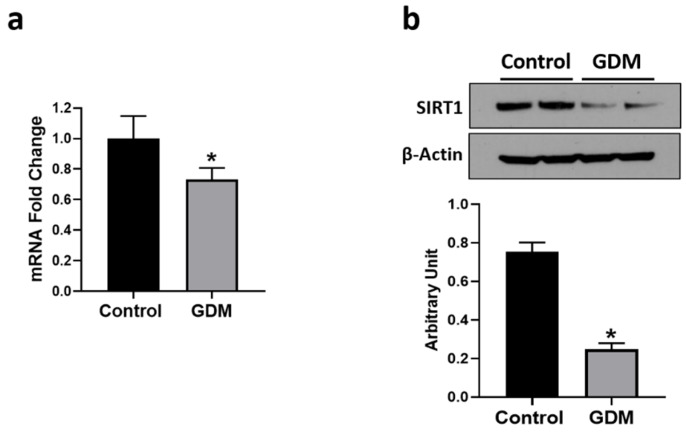
Changes in mRNA and protein levels of SIRT1 in the trophoblasts exposed to control and GDM conditions. Primary human trophoblasts were cultured in standard conditions or with medium containing 25-mmol/L glucose and 10^−7^-mol/L insulin for 72 h. (**a**) Real-time PCR was used to measure SIRT1 mRNA levels. (**b**) Western blotting was used for the quantification of SIRT1 protein. Representative blots for SIRT1 is shown at the top; blot density obtained from densitometric scanning of SIRT1 normalized to β-actin is shown at the bottom. Values are given as means ± SD three independent experiments. * *p* < 0.05 vs. control.

SIRT1 is an enzyme that regulates cellular lipid metabolism. SIRT1 expression and activity are increased under fasting or calorie restricted conditions and attenuated by a high energy environment [42,43]. SIRT1 mRNA and protein levels were significantly reduced by 30% and 68%, respectively, in the trophoblasts from GDM conditions relative to controls (*p* < 0.05) (Figure 5a,b), which indicates that IR reduces SIRT1 expression in trophoblasts. 

### 3.6. Effect of SIRT1 Expression on DHA Transport 

**Figure 6:**
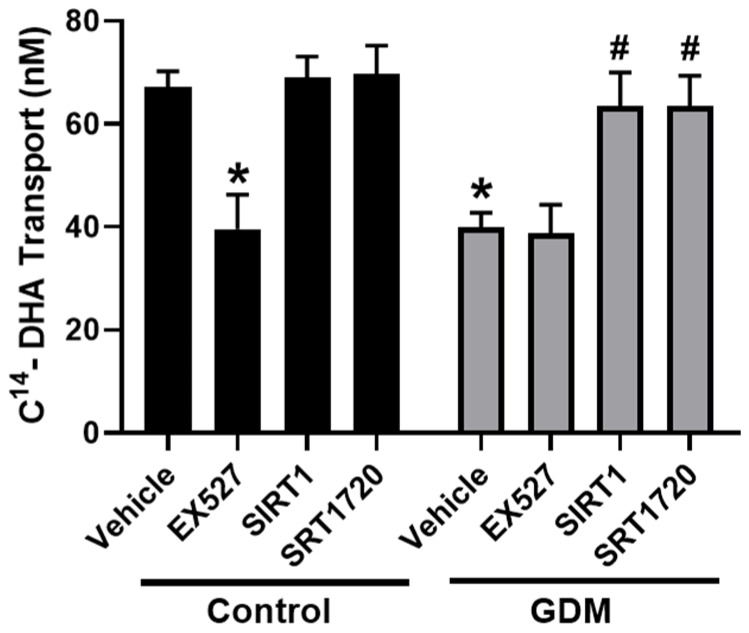
Effect of SIRT1 on DHA transfer through the trophoblasts exposed to control and GDM conditions. Primary human trophoblasts were cultured in Transwell inserts in standard conditions or with medium containing 25-mmol/L glucose and 10^−7^-mol/L insulin for 72 h. Cells cultured in control conditions were treated with SIRT1 inhibitor (EX527, 1 µmol/L) for 24 h. Cells cultured under GDM conditions were treated with recombinant SIRT1 (50 ng/L) or SIRT1 activator (SIRT1729, 1 µmol/L) for 24 h. Transfer of 14C-DHA (1 μCi/mL) from the apical to the basal chamber was measured using liquid scintillation counting. Data are expressed as mean ± SD of three independent experiments. * *p* < 0.05 vs. vehicle in control conditions. # *p* < 0.05 vs. vehicle in GDM conditions.

We next studied the regulatory effect of SIRT1 on DHA transport capacity in trophoblasts. To verify the role of decreased SIRT1, we treated trophoblasts cultured under standard conditions with a SIRT1 inhibitor (EX527, 1 µmol/L). Significantly reduced levels of DHA transport across the trophoblasts were found in EX527-treated trophoblasts (41% decrease) compared with the vehicle-treated trophoblasts in control conditions (Figure 6), indicating that SIRT1 facilitates DHA transport. EX527 treatment did not alter DHA transport in trophoblasts from GDM conditions.

### 3.7. Effect of SIRT1 on DHA Transport in GDM Conditions

To further study the effect of SIRT1 in trophoblasts from GDM conditions, we treated these cells with the SIRT1 activator (SIRT1720, 1 µmol/L) and recombinant SIRT1 (50 ng/L). As shown in Figure 6, significantly increased DHA transport levels were found in SIRT1720-treated trophoblasts from GDM conditions. Similarly, enhanced DHA transport was observed in recombinant SIRT1-treated cells (Figure 6). SIRT1 and SRT1720 did not significantly alter DHA transport in control trophoblasts. Together, these studies demonstrate that SIRT1 restores DHA transfer across trophoblasts during GDM conditions. 

## 4. Discussion

This study, for the first time, investigated the impact of insulin-resistant GDM conditions on DHA transport across primary human (syncytio)trophoblasts. Our study demonstrates that elevated glucose and insulin levels decrease DHA transfer across trophoblasts despite increased triglyceride accumulation and increased expression of fatty acid transporter genes. The reduced DHA transfer was associated with decreased SIRT1 expression in the trophoblasts. Inhibition of SIRT1 decreased DHA transport in control trophoblasts, while activation of SIRT1 restored DHA transfer in trophoblasts exposed to GDM conditions. Therefore, we suggest that SIRT1 plays an important role in the regulation of DHA transport across the placenta, and the suppressed SIRT1 expression and function might contribute to reduced transplacental transfer of DHA during GDM pregnancies. 

GDM is characterized by IR in peripheral tissues [44], but few studies have examined how IR could alter placental nutrient transport. The patients with GDM have severe glucose intolerance with associated maternal hyperglycemia and insulinemia [45]. During GDM, the glucose transport through the placenta is unaffected as GLUT-1, the main carrier of glucose through the placenta, acts independent of insulin [46]. Thus, maternal hyperglycemia leads to fetal hyperglycemia, which then results in fetal hyperinsulinemia [47].

In contrast, knowledge on adaptations of placental fatty acid metabolism in response to GDM is sparse. Insulin receptors are expressed in the placenta, and maternal insulin can activate the insulin receptor-related signaling, thus affecting placental metabolism [48]. In this study, exposure of trophoblasts to elevated glucose and insulin levels to mimic the state of the cells in the GDM placentas, induced an effect that is reminiscent of IR with decreased IRS-1 and increased IGF1 and leptin mRNA, similar to that established in BeWo trophoblast cells [41]. Our finding of decreased DHA transport through trophoblasts exposed to GDM conditions indicates that IR can directly compromise the ability of trophoblasts to transport DHA through them. These findings corroborate previous reports relating to reduced DHA transport in placentas from women with GDM [15,16,17,18,19,20,21,22,23].

Trophoblasts cultured in GDM conditions had higher triglyceride content, suggesting that under such circumstances, there is increased fatty acid esterification for the storage of fatty acids in the form of triglycerides. We found a higher expression of CD36 in trophoblast from GDM conditions. The higher CD36 indicates increased uptake of DHA and other fatty acids from the maternal circulation into the trophoblasts [49]. The fatty acids that are present in the serum used for culturing could have been taken up by the trophoblasts and esterified for storage in the form of triglycerides in GDM conditions. The finding of increased CD36 expression and higher triglyceride content in trophoblasts exposed to GDM conditions is similar to that observed in the placenta from GDM patients [27,50,51].

The expression of FABP3 and FABP4 were increased in trophoblasts from GDM conditions. FABP3 and FABP4 are among the major fatty acid-binding proteins that function as intracellular lipid chaperones to shuttle fatty acids from the maternal side of the plasma membrane to target organelles (i.e., to mitochondria for oxidation and for storage as lipid droplets) or to the basal side of the membrane for delivery to the fetus [52,53,54]. FABP3 and FABP4 have been shown to have a higher binding affinity to DHA and other long-chain polyunsaturated fatty acids [55,56]. The knockdown of FABP3 decreased DHA transport across BeWo trophoblasts [52]. Thus, increased expression of FABP3 is expected to enhance the transport of DHA across the trophoblast cells. However, it is surprising why DHA transport decreased despite increased FABP3 and FABP4 expression in trophoblasts from GDM conditions. Oxidative stress is known to reduce the binding affinity of FABP4 to DHA [55]. Since GDM conditions are known to induce oxidative stress, this could compromise the ability of FABP3 and FABP4 to bind with DHA and shuttle them to the fetal side. Another possibility is that increased FABP3 and FABP4 may preferentially direct fatty acids for deposition into lipid droplets. This notion is supported by the reports of FABP4 promoting esterification and lipid accumulation in trophoblasts [57], macrophages [58], and cardiomyocytes [59]. Another underlying question is what contributes to the increased FABP3 and FABP4 expression in trophoblasts from GDM conditions. FABP4 promoter has a binding site for insulin, fatty acids, and hypoxia-inducible factor [60,61], and these are all increased in GDM conditions [45,62,63], possibly contributing to FABP3 and FABP4 promoter activation and upregulation. Consistent with our findings, increased placental FABP3 and FABP4 expression and a strong correlation of circulatory FABP4 levels with GDM have been reported [27,64].

Varied levels of FATP expression have been reported in the placenta of GDM pregnancies [27,65]. The finding of unaltered FATP1, FATP2, FATP4, and FATP6 expression in trophoblasts exposed to GDM conditions suggest that changes solely to the glucose and insulin environment are unlikely to alter the expression of these transporters. Rather, any such alterations could be the consequence of a compensatory response that occurs in an in vivo setting or maybe a response to composite changes to the metabolic milieu, including altered cytokine profile reflective of systemic inflammation in GDM [66]. Collectively, our findings of increased fatty acid transporter expression (CD36, FABP3, and FABP4), enhanced triglyceride accumulation, and concomitant reduction in DHA transport in GDM conditions suggest that the DHA is taken up by the trophoblasts and retained after esterification into triglycerides instead of being transferred to the fetus. Consistent with this notion, the placenta of GDM women are found to have higher DHA and triglycerides [51].

In search of the mechanism for the decreased DHA transport through the trophoblasts, we identified that SIRT1 expression was significantly reduced in trophoblasts exposed to GDM conditions. There have not been any studies on alterations of placental SIRT1 expression in the setting of GDM, which would be interesting to examine in the future. SIRT1 expression is highly regulated by energy status. Calorie restriction increases SIRT1 expression, and high energy status decreases SIRT1 expression [67,68]. Thus, the hyperglycemic GDM conditions could contribute to the decreased SIRT1 expression, as reported in macrophages [69]. However, the novel finding of our study is that the SIRT1 inhibitor decreased DHA transfer through trophoblasts cultured under physiological conditions. This finding suggests that SIRT1 has a direct regulatory role in controlling DHA transport across the placenta during normal pregnancy. The decreased SIRT1 expression in trophoblasts from GDM conditions indicates that the reduced DHA transfer across trophoblasts in GDM conditions could be consequent to decreased SIRT1 expression and function. Indeed, the primary role of SIRT1 mediating DHA transfer is further strengthened by our data that treatment with the recombinant SIRT1 and SIRT1 activator resulted in the enhancement of DHA transport across trophoblasts exposed to GDM conditions. It is important to note that Sirt1-null mice embryos are growth restricted with abnormalities in fetal heart development [37]. Although it is tempting to postulate that abnormal placental lipid metabolism and decreased DHA transport to the fetus in the Sirt1-null embryos as a probable cause of fetal growth restriction, additional in vivo studies are required to determine the extent to which suppressed SIRT1 expression and function contributes to this phenotype. Additionally, the exact mechanism by which SIRT1 regulates DHA transport and if SIRT1 also regulates the transport of other fatty acids needs to be clarified in the future. 

## 5. Conclusions

In conclusion, the present study demonstrates that high glucose and insulin levels of the GDM environment decrease DHA transport through the trophoblasts via decreased SIRT1-mediated signaling, providing a molecular mechanism linking gestational diabetes and decreased transplacental DHA transport. Although caution should always be observed in extrapolating the findings of in vitro studies directly to humans, the present result provides a mechanistic understanding worthy of investigation in in vivo studies. Strategies that target activation of SIRT1 could have a role mitigating fatty acid dyslipidemia in pregnancies complicated by gestational diabetes.

## Figures and Tables

**Table 1:** Quantitative real-time PCR primer sequence.

Gene	Forward	Reverse
IRS-1	TGGGGACTACAAGGTAGGGG	ATGCCACCTGGCTGAATGAA
IGF-1	AGAGCCTGCGCAATGGAATA	GAGATGCGAGGAGGACATGG
IGF-2	ACACCCTCCAGTTCGTCTGT	GGGGTATCTTGGGGAAGTTGT
Leptin	GCTGGAGAAGCTCACCCAAT	CAAAGTGCAAGCAGGGTTCC
FAT/CD36	TTGGAGACCTGCTTATCCAGAAGACAATT	AAACTGTCTGTAAACTTCTGTGCCTGTTTTAAC
FAT/P1	TGTCTCATCTATGGGCTGACAGTCG	GTACTGAACCACCGTGCAGTTGTACT
FAT/P2	CGTCACTGTCATTCAGTATATCGGTGAAC	ATTTCCCAGTGCCAGTCTCACTTTATGA
FAT/P4	TCTTTGGCAGCGAAATGGCCTCAG	AGAGCAGAAGAGGCTGAGCGA
FAT/P6	TGGAGAACTTTGTCGCTACCTTTGCAAA	CACTCCGTATGCCATTTCCAATTGCC
FABP3	TGAGACAACAGCAGATGACAGGAAGG	ATCAATTAGCTCCCGCACAAGTGTG
FABP4	GGTACCTGGAAACTTGTCTCCAGTGA	TCACATCCCCATTCACACTGATGATCATG
SIRT1	TCTTCCCTCAAAGTAAGACCAGTAGCACTA	CTACATCAAAATGCAGATGAGGCAAAGGT
